# Editorial: Metabolic Disorders Associated With Autism Spectrum Disorders: Approaches for Intervention

**DOI:** 10.3389/fnins.2021.809978

**Published:** 2021-12-02

**Authors:** Joana M. Gaspar, Humberto M. Carvalho, Alberto Camacho-Morales

**Affiliations:** ^1^Laboratory of Bioenergetics and Oxidative Stress, Department of Biochemistry, School of Biological Sciences, Federal University of Santa Catarina, Florianópolis, Brazil; ^2^Department of Physical Education, School of Sports, Federal University of Santa Catarina, Florianópolis, Brazil; ^3^Department of Biochemistry, College of Medicine, Autonomous University of Nuevo León, Mexico, Mexico; ^4^Neurometabolism Unit, Center for Research and Development in Health Sciences Autonomous University of Nuevo León, Mexico, Mexico

**Keywords:** autism spectrum—disorder, gut-brain axis, physical exercise, GABA, sensory perception, cholinergic system, inborn errors of metabolism

Autism spectrum disorder (ASD) is a neurodevelopmental disease that impairs efficient responses to external sensory-stimulus and defective social interaction. ASD affects 1% of the population and has different severity degrees (Maenner et al., 2020). The pathogenesis of ASD is not completely understood, but involves a combination of genetic, environmental factors and immune dysfunction. Associated with immune system activation, ASD results from the central nervous system derangements due to chronic inflammatory reactions (Toscano et al., 2021). In addition, ASD is associated with several comorbidities, including gastrointestinal disturbances, feeding problems, selective eating behaviors, and obesity (McElhanon et al., 2014; Granich et al., 2016). In this context, metabolic, inflammatory, and brain diseases are likely mutually linked; however, a clear mechanistic understanding has remained elusive.

The major research goals of this collection were to find causes, examine developmental trajectories of the disorder, and develop new or improved time-dependent interventions or preventions to contribute to the overall health and well-being of ASD individuals. Specifically, improving early and accurate strategies for diagnosis, understanding the role of the endocrine, metabolic, immunologic pathways, and neuropathology of ASD, and testing potential dietary and behavioral interventions for ASD.

Gut peptide hormones have been found across different brain regions, and clinical studies have proposed that many of those hormones are involved in autism-related social, emotional, and cognitive deficits. Qi and Zhang reviewed and summarized major findings from clinical and animal studies showing the role of gut peptide hormones (CCK, VIP, secretin, ghrelin, and PACAP) in mediating autism-related neurological functions and their potential implications in autism pathogenesis and autism-related neurological functions (Qi and Zhang). The authors also discussed the potential use of these gut hormones as therapeutic targets to manage ASD symptoms (Qi and Zhang).

Preclinical and clinical studies have shown that ASD individuals display alterations of the gut microbiota and their metabolite production, which can be used as biomarkers. Biomarkers related to the gut-brain axis would greatly value the diagnosis, development, and follow-up of potential therapies for patients with ASD. The link between ASD and the gastrointestinal microbiome, their metabolites, and the use of metabolites as biomarkers (such as short-chain fatty acids, phenolic compounds, and free amino acids) was reviewed (Garcia-Gutierrez et al.). The production of metabolites and neurotransmitters by gut microbiota can stimulate the immune system and influence the central nervous system by vagal stimulation. Thus, therapies-based interventions that modulate gut microbiota (probiotics, prebiotics, and fecal microbiota transplantation therapy) have arisen as a potential treatment to ameliorate ASD symptoms (Garcia-Gutierrez et al.).

A significant number of ASD cases could be associated with various metabolic abnormalities, some of them identifiable only through untargeted metabolomic profiling, emphasizing the causal role of inborn errors of metabolism in ASD. Symptoms of ASD can be present in many inborn errors of metabolism but rarely occur in isolation. Žigman et al. give an overview of inborn errors of metabolism characterized by symptoms of ASD. The authors also proposed a diagnostic approach to assess cases in clinical practice and possible specific therapies. The authors briefly describe etiology, clinical presentation, and therapeutic principles, for several inborn errors of metabolism in differential diagnosis of ASD (disorders of the amino acid metabolism, mitochondrial disorders, cholesterol biosynthesis defects, neurotransmitter disorders, folate metabolism, lysosomal storage disorders, among others). As always, detailed medical history and clinical examination, including detailed neurological examination, should be a basis for planning focused diagnostic work-up of patients with ASD (Žigman et al.).

ASD individuals have deficiencies in motor skills. Physical activity reduces maladaptive behaviors, such as stereotypes, and positively affects social skills in young children and adolescents with autism. The association of classical pharmacological treatments with exercise and other physical activities to intervention programs with children with ASD may be beneficial. Sefen et al. reviewed the exercise-therapy as an appropriate therapeutic strategy for improving the quality of life in ASD (Sefen et al.). The authors addressed the beneficial effects of structured physical activities for children with ASD and discussed the importance of parental involvement in physical activity (Sefen et al.). The authors point out that to build a physical activity program for children with ASD, it is important to address multiple factors to decide the most appropriate elements of the program, such as individual vs. group intervention and the organization of the program. Each person with autism has a highly individualized set of symptoms and characteristics for which highly individualized physical activity programs are warranted (Sefen et al.).

Sensory hyper-responsiveness is included in the restricted interests and repetitive behaviors central to an ASD diagnosis. Studies have observed that altered γ-aminobutyric acid (GABA) neurotransmission is a central characteristic of the neurophysiology of ASD and may explain the sensory abnormalities. The group of Masakazu Ide assessed whether GABA concentrations in specific brain areas [primary visual cortex, sensorimotor cortex, supplementary motor area (SMA), and ventral premotor cortex (vPMC)] were associated with different domains of abnormal sensory experiences in individuals with ASD (Umesawa et al.). Also, using ^1^H magnetic resonance spectroscopy, they addressed whether GABA levels were associated with abnormal sensory responses in ASD (Umesawa et al.). They observed a negative association between left vPMC GABA and the severity of sensory hyper-responsiveness. The authors suggest that reduced inhibitory neurotransmission in a higher-order motor area that integrates multiple sensory modalities may underlie sensory hyper-responsiveness in ASD (Umesawa et al.).

A final article aimed to identify the relationship between ASD and cholinergic signaling using a systems biology model. Acetylcholine plays a key role in cognitive function, memory, learning, and sensory processing signal transduction. Autism genome-wide association studies (GWAS) have provided estimates of the overlap of autism-related gene sets with those involved in sensory processing and cholinergic signal transmission. The authors evaluated the relationship between ASD and choline intake (Olson et al.). This article documented a gene set enrichment analysis results focusing on shared gene ontologies between an autism-associated gene set obtained through meta-analysis of GWAS and a gene set centering on cholinergic function (Olson et al.).

## Author Contributions

All authors listed have made a substantial, direct, and intellectual contribution to the work and approved it for publication.

## Conflict of Interest

The authors declare that the research was conducted in the absence of any commercial or financial relationships that could be construed as a potential conflict of interest.

## Publisher's Note

All claims expressed in this article are solely those of the authors and do not necessarily represent those of their affiliated organizations, or those of the publisher, the editors and the reviewers. Any product that may be evaluated in this article, or claim that may be made by its manufacturer, is not guaranteed or endorsed by the publisher.
